# 

**Published:** 1899-01

**Authors:** 


### BOOKS RECEIVED.

The Origin and Progress of Renal Surgery, with special reference to Stone in the Kidney and Ureter, and to the Surgical Treatment of Calculous Anuria; being the Hauterian Lectures for 1898, together with a critical examination of suppurietal injuries of the ureter. Henry Morris, M. A., M. B. Lond.; F. R. C. S.; Senior Surgeon to the Middlesex Hospital; Examiner in Surgery in the University of London; Member of the Council and Chairman of the Court of Examiners of the Royal College of Surgeons of England; Hauterian Professor of Surgery and Pathology, Royal College of Surgeons of England; Honorary Member of the

Medical Society of the County of New York. Philadelphia: P. Blakiston's Son & Co. 1898. Price, $2.00.

The Phonendoscope and its Practical Application. By Aurelio Bianchi, M. D., Professor of Preparatory Clinical Medicine and of Pathology, at Parma, Italy. With 37 illustrations. Translated by A. George Baker, A. M., M. D.; Physician-in-Chief of the Chinese Medical Dispensary, Philadelphia. Octavo, pp. 77. Philadelphia: George P. Pilling & Son. 1898. Price, 50 cents.

Acromegaly. An Essay to which was awarded the Boylston Prize of Harvard University for the year 1898. By Guy Hinsdale, A. M., M. D. Fellow of the College of Physicians of Philadelphia, and of the American Academy of Medicine; Member of the American Neurological and Climatological Associations; Assistant Physician to the Orthopedic Hospital and Infirmary for Nervous Diseases, and to the Presbyterian Hospital, Philadelphia. Detroit: Reprinted from *Medicine* William M. Warner, Publishers. 1898. Price, $1.00.

The Practitioner's Manual. A condensed system of medical diagnosis and treatment. By Charles Warrenne Allen, M. D.; Consulting Genito-Urinary Surgeon to the City (Charity) Hospital; Consulting Dermatologist to the Randall's Island Hospital; Attending Surgeon to the Good Samaritan Hospital, etc., etc. 8vo, pp. 85. New York: William Wood & Co. 1898.

A Manual of Physiology, with practical exercises. By G. N. Stewart, M. A. D. Sc., M. D., Edin.; D. P. H., Camb.; Professor of Physiology in the Western Reserve University, Cleveland, etc., etc. With numerous illustrations, including five colored plates. Third edition. Small 8vo., pp. 848. Philadelphia: W. B. Saunders. 1898. Price, $3.75.

The Sexual Instinct: Its Use and Dangers as Affecting Heredity and Morals. By James Foster Scott, B A. (Yale University), M. D., C. M. (Edinburgh University): Late Obstetrician to Columbia Hospital for Women and Lying-in Asylum, Washington, D. C., etc. Octavo, pp. 436. New York: E. B. Treat & Co. 1899. Price, $2.00.

An Atlas of Bacteriology, containing 111 original photomicrographs with explanation text. By Charles Slater, M. A., M. B., M. R. C. S., Eng.; F. C. S., Lecturer on Bacteriology, St. George's Medical School; and Edmund J. Spilta, L. R. C. P., London; M. R. C. S., Eng.; F. R. A. S.; Formerly Demonstrator of Anatomy, St. George's Hospital Medical School, London. The Scientific Press Ltd., 28 Southampton Street, Strand. 1898. Price, $2.50.

Transactions of the American Gynecological Society, Vol. XXIII., for the year 1898. Small 8vo, pp. 491. Philadelphia: Wm. J. Dornan, Printer. 1898.

Transactions of the American Otological Society. Thirty-first Annual Meeting, New London, Conn., July 19, 1898. Vol. VII., Part —. New Bedford, Mass.: Mercury Publishing Co. 1898.

State Board of Health of Pennsylvania. Thirteenth Annual Report, for the year 1897. In two volumes. Wm. Stanley Ray, State Printer. 1898.

A Text-Book of Obstetrics. By Barton Cooke Hirst, M. D., Professor of Obstetrics in the University of Pennsylvania. With 653 illustrations. Octavo, pp. 846. Philadelphia: W. B. Saunders. 1898. Price, cloth, $5.00; sheep, $6.00.

A Manual of Bacteriology. By Herbert U. Williams, Professor of Pathology and Bacteriology, Medical Department, University of Buffalo. 8vo, pp. 263. With 78 illustrations. Philadelphia: P. Blakiston's Son & Co. 1898. Price, $1.50.

A Syllabus of Materia Medica. Compiled by Warren Coleman, M. D., Instructor in Clinical Medicine and Materia Medica in Cornell University, Medical Department. One volume. 16mo, 175 pages. Polished buckram, $1.00 net. New York: William Wood & Co. 1899.

